# Versorgungswirklichkeit der urologischen Endoprothetik in Deutschland von 2006 bis 2016

**DOI:** 10.1007/s00120-021-01444-5

**Published:** 2021-01-22

**Authors:** Martin Baunacke, Christer Groeben, Angelika Borkowetz, Annemarie Uhlig, Marianne Leitsmann, Björn Volkmer, Christian Thomas, Johannes Huber

**Affiliations:** 1 Klinik und Poliklinik für Urologie, Universitätsklinikum Carl Gustav Carus an der Technischen Universität Dresden, Fetscherstraße 74, 01307 Dresden, Deutschland; 2 Klinik für Urologie, Universitätsmedizin Göttingen, Georg-August-Universität, Göttingen, Deutschland; 3 Klinik für Urologie, Klinikum Kassel, Kassel, Deutschland

**Keywords:** Sphinkterprothese, Penisprothese, Inkontinenz, Erektile Dysfunktion, Versorgung, Sphincter prosthesis, Penile prosthesis, Incontinence, Erectile dysfunction, Health care research

## Abstract

**Hintergrund:**

Die Behandlung von Harninkontinenz und erektiler Dysfunktion verbessert die Lebensqualität vieler Patienten. Insbesondere die Endoprothetik mit Sphinkter- und Penisprothesen erzielt hierbei sehr gute Ergebnisse, wenn konservative Therapieoptionen ausgeschöpft sind. Ziel dieser Studie ist eine Darstellung der Entwicklung und aktuellen Versorgungslage der Sphinkter- und Penisprothesenimplantation in Deutschland.

**Material und Methoden:**

Wir führten eine Analyse der Diagnosis-Related-Groups-Abrechnungsdaten in Deutschland im Zeitraum von 2006 bis 2016 durch. Die Versorgungslage im Jahr 2016 beschrieben wir auf Basis der Qualitätsberichtsdaten der deutschen Krankenhäuser.

**Ergebnisse:**

Von 2006 bis 2012 stieg die Zahl der implantierten Sphinkterprothesen in Deutschland von 739 auf 1112 (*p* < 0,001) und die Zahl der implantierenden Kliniken von 129 auf 206 (*p* < 0,001). Von 2012 bis 2016 fielen die Fallzahlen auf 980 und die Zahl der Kliniken auf 198. Im Jahr 2016 implantierten 168 (88 %) urologische Kliniken 1–9 Sphinkterprothesen und 23 (12 %) Kliniken ≥ 10 Sphinkterprothesen. Die 10 Top-Kliniken (≥20 Sphinkter) implantierten 34 % (283/839) aller Sphinkter. Von 2006 bis 2013 stieg die Zahl der implantierten Penisprothesen kontinuierlich von 263 auf 503 (*p* < 0,001) sowie die Zahl der implantierenden Kliniken von 71 auf 107 (*p* < 0,001). Von 2013 bis 2016 stagnierte die Fallzahl (*p* = 0,9) und die Zahl der implantierenden Kliniken (*p* = 0,5). Der Anteil implantierter Penisprothesen im Rahmen von Geschlechtsumwandlungen stieg von 17 % im Jahr 2006 auf 25 % im Jahr 2016 (*p* = 0,03). Im Jahr 2016 implantierten 83 (85 %) urologische Kliniken 1–6 Penisprothesen und 14 (15 %) Kliniken ≥ 7 Prothesen. Die 7 Top-Kliniken (≥20 Prothesen/Jahr) implantierten 232/448 (52 %) der Prothesen.

**Diskussion:**

Der Versorgungsstand der urologischen Endoprothetik in Deutschland zeigt eine deutliche Zentrenbildung, aber auch eine große Zahl von Kliniken mit geringer Fallzahl. Seit 2012/2013 zeigt sich eine Stagnation der Fallzahlen von Penis- und Sphinkterprothesenimplantationen, die in Zusammenschau mit den Prostatektomiefallzahlen eine Unterversorgung vermuten lässt.

**Zusatzmaterial online:**

Die Online-Version dieses Artikels (10.1007/s00120-021-01444-5) enthält weitere Tabellen zu Fallzahlen von Sphinkterprothesen und Penisprothesenimplantationen. Beitrag und Zusatzmaterial stehen Ihnen auf www.springermedizin.de zur Verfügung. Bitte geben Sie dort den Beitragstitel in die Suche ein, das Zusatzmaterial finden Sie beim Beitrag unter „Ergänzende Inhalte“.

## Abstract (en)

**Background:**

Treating urinary incontinence and erectile dysfunction improves quality of life for many patients. In particular, sphincter and penile prostheses achieve very good results when conservative therapy options are exhausted. The aim of this study is to present the development and current state of sphincter and penile prosthesis implantation in Germany.

**Materials and methods:**

We carried out an analysis of the Diagnosis Related Groups billing data in Germany from 2006–2016. We described the state of care in 2016 based on the German hospitals’ quality reports.

**Results:**

Between 2006 and 2012 implantations of sphincter prostheses in Germany increased from 739 to 1112 (*p* < 0.001), the amount of implanting hospitals also increased from 129 to 206 (*p* < 0.001). From 2012–2016, the number of cases decreased to 980 and the number of hospitals to 198. In 2016, 168 (88%) urological hospitals implanted 1–9 sphincter prostheses and 23 (12%) hospitals implanted ≥ 10 sphincter prostheses. The top 10 hospitals (≥20 sphincters) implanted 34% (283/839) of all sphincters. Between 2006 and 2013 the number of implanted penile prostheses continuously increased from 263 to 503 (*p* < 0.001), the number of implanting hospitals from 71 to 107 (*p* < 0.001). From 2013–2016, the number of cases (*p* = 0.9) and the number of implanting hospitals (*p* = 0.5) stagnated. The proportion of penile prostheses implanted as part of gender reassignment surgery increased from 17% in 2006 to 25% in 2016 (*p* = 0.03). In 2016, 83 (85%) urological hospitals implanted 1–6 penile prostheses and 14 (15%) hospitals implanted ≥ 7 prostheses. The 7 top hospitals (≥20 prostheses/year) implanted 232/448 (52%) of the prostheses.

**Conclusions:**

The current state of urological endoprosthetics in Germany shows a small number of high-volume centers, but also a large number of hospitals with a small number of cases. Since 2012/2013, there has been a stagnation in the number of cases of penile and sphincter prosthesis implantations. In view of the number of radical prostatectomy cases, this development suggests an undersupply.

## Einleitung

Sphinkter- und Penisprothesen sind die komplexesten Systeme in der urologischen Endoprothetik. Trotz ihres Alters – beide Systeme wurden Anfang der 1970er-Jahre entwickelt – stellen Sie immer noch den Goldstandard der Therapie in ihren jeweiligen Einsatzgebiet dar: Harninkontinenz und erektile Dysfunktion.

Sphinkterprothesen werden bei ausgeprägter Belastungsinkontinenz eingesetzt, bei der konservative und medikamentöse Maßnahmen versagt haben und andere operative Verfahren wie Schlingen oder Systeme aufgrund des hohen Grades der Belastungsinkontinenz nicht in Frage kommen. Bei Sphinkterprothesen können eine oder zwei Manschetten um die Harnröhre gelegt werden; insbesondere bei Frauen erfolgt die Anlage um den Blasenhals. Die Sphinkterprothese stellt den Goldstandard bei der Therapie der männlichen Belastungsinkontinenz nach radikaler Prostatektomie (RP) dar und erreicht hier hohe Erfolgsraten zwischen 59–90 % [1–3]. Nachteile der Sphinktersysteme sind die nicht zu vernachlässigenden Komplikations- und Revisionsraten sowie die begrenzte Haltbarkeit [4]. Die Revisionsrate sinkt mit zunehmender operativer Erfahrung [5].

Penisprothesen sind Schwellkörperimplantate und finden ihren Einsatz nach zahlreichen Therapiemöglichkeiten der erektilen Dysfunktion wie oraler und urethraler Pharmakotherapie, Schwellkörperautoinjektion oder Vakuumpenispumpen. Ein weiterer relevanter Anwendungsbereich ist der Einsatz in der operativen Geschlechtsangleichung. In kleinerer Zahl werden Penisprothesen auch im Rahmen von Penisrekonstruktionen nach Amputationen bei Unfällen oder Peniskarzinomen implantiert [6]. Bei Penisprothesen wird zwischen semirigiden und hydraulischen Prothesen unterschieden. Trotz der Radikalität des Verfahrens ist die Penisprothese der Goldstandard nach Versagen konservativer Therapien. In diesem Patientenkollektiv mit hohem Leidensdruck erreicht die Penisprothese deutlich höheren Zufriedenheitsraten als die Pharmakotherapie [1]. Auch die Penisprothesenimplantation weißt eine relevante Komplikationsrate auf [7], welche durch operative Erfahrung gesenkt werden kann [8].

Trotz guter Erfolgsraten von Sphinkter- und Penisprothesen konnten wir aktuell Anhaltspunkte für ein relevantes Versorgungsdefizit in der Behandlung von Patienten mit Inkontinenz bzw. erektiler Dysfunktion nach RP in Deutschland finden [9]. Daher ist es Ziel dieser Studie, die Versorgungswirklichkeit der Sphinkter- und Penisprothesenimplantation in Deutschland zu analysieren.

## Material und Methoden

Wir führten eine Analyse der Diagnosis Related Groups(DRG)-Abrechnungsdaten in Deutschland im Zeitraum von 2006 bis 2016 über das Statistische Bundesamt (Destatis) durch. Hierbei erfolgte für Sphinkterprothesen die Abfrage der jährlichen Patientenzahl für folgende Operationen- und Prozedurenschlüssel(OPS)-Codes: 5‑597-00 (Implantation bulbär, 1 Cuff), 5‑597-01 (Implantation bulbär, 2 Cuffs) und 5‑597-02 (Implantation am Blasenhals). Für den OPS-Code 5-597‑0 (Implantation) erfolgte zusätzlich die Abfrage der Zahl der abrechnenden Kliniken und des Patientenalters. Für Penisprothesen erfolgte die Analyse folgender OPS-Codes: 5‑649‑5 (Implantation einer Penisprothese), 5‑649-50 (Implantation semirigide Prothese) und 5‑649-51 (Implantation hydraulische Prothese). Für die OPS 5-649‑5 wurden zusätzlich die Zahl der abrechnenden Kliniken, das Patientenalter und die Verknüpfung mit der ICD(Internationale statistische Klassifikation der Krankheiten) F64 (Störung der Geschlechtsidentität) ermittelt. Zur weiteren Einordnung der Fallzahlen erfolgte die Abfrage der OPS 5-604* (radikale Prostatovesikulektomie) [10].

Die Beschreibung der Versorgung im Jahr 2016 erfolgte mit o.g. Codes anhand der Qualitätsberichtsdaten (QB-Daten) der deutschen Krankenhäuser für das Jahr 2016. Die Auswertung der Qualitätsberichtsdaten erfolge mit der Software QB-Monitor 2016 (Lutum + Tappert DV-Beratung GmbH, Bonn). Aus Datenschutzgründen wird in den QB-Daten zu ICD- und OPS-Kennziffern mit einer Anzahl von 1, 2 oder 3 die tatsächliche Zahl nicht ausgewiesen. Im QB-Monitor wird in diesen Fällen die Zahl 1 angegeben. Die ermittelten Kliniken wurden bezüglich der Bettenzahl des Krankenhauses, der Bettenzahl der urologischen Klinik, der Stadtgröße und der RP-Fallzahl klassifiziert. Außerdem ergänzten wir, ob es sich um eine Universitätsklinik und ein zertifiziertes Prostatakarzinomzentrum handelte. Bei der Sphinkterprothesenimplantation erfolgte folgende Einteilung: Kliniken mit geringer Fallzahl (1–9 Sphinkterprothesen) und hoher Fallzahl (≥10 Sphinkterprothesen). Bei der Penisprothesenimplantation erfolgte folgende Einteilung: Kliniken mit geringer Fallzahl (1–6 Penisprothesen) und hoher Fallzahl (≥7 Penisprothesen). Die 10 Top-Kliniken bei Sphinkterprothesen implantierten ≥ 20 Sphinkterprothesen 2016. Die 7 Top-Kliniken bei Penisprothesen implantierten ≥ 20 Penisprothesen 2016. Die Kartendarstellungen erfolgten mit der Software easymap© office (Lutum + Tappert DV-Beratung GmbH, Bonn).

Da alle verwendeten Daten anonymisiert sind, war kein Ethikvotum erforderlich. Die Datenanalyse erfolgte mit dem χ^2^-Test, Mann-Whitney-U-Test und linearer Regressionsanalyse (Signifikanzniveau *p* = 0,05). Die statistische Auswertung erfolgte mit „IBM SPSS Statistics 27“ (Armonk, NY, USA).

## Ergebnisse

### Sphinkterprothetik

**Figure 1:**
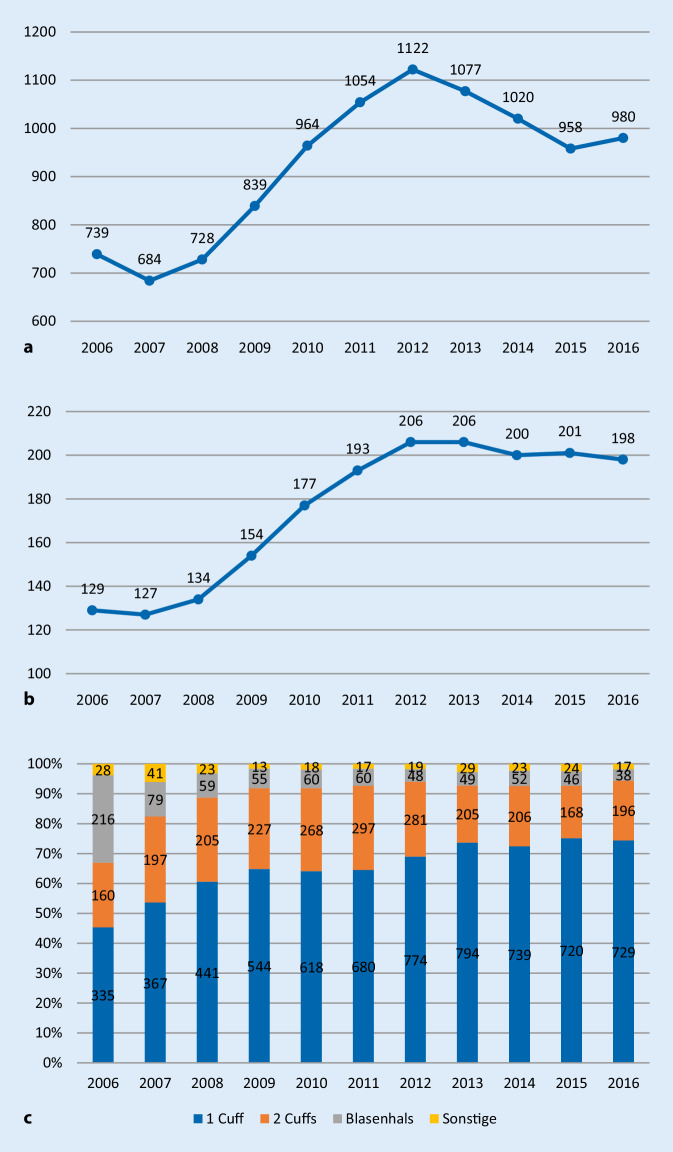

Von 2006 bis 2012 zeigte sich ein kontinuierlicher Anstieg der implantierenden Kliniken (+15/Jahr, *p* < 0,001) und der Gesamtfallzahl (+76/Jahr, *p* < 0,001). Ab 2012 nahm die Zahl der Implantationen von 1122 auf 980 Implantationen ab (−40/Jahr, *p* = 0,02) und die Anzahl der Kliniken sank von 206 auf 198 (−2/Jahr, *p* = 0,03). Der Anteil an Frauen bei Sphinkterimplantationen blieb zwischen 2006 bis 2016 mit 2–5 % konstant. Der Anteil von Sphinktern mit einem Cuff nahm von 2006 bis 2016 von 45 % (335/739) auf 74 % (729/980) zu (*p* < 0,001), während der Anteil der Doppel-Cuff-Prothesen leicht sank – von 22 % (160/739) 2006 bzw. 29 % (197/684) 2007 auf 20 % (196/980) 2016 (*p* = 0,02). Auch der Anteil von Sphinktern mit Blasenhalsmanschette sank deutlich von 29 % (216/739) auf 4 % (38/980; *p* = 0,02; Abb. 1). Der Anteil männlicher Patienten bei der Implantation von Sphinktern mit Blasenhalsmanschette nahm ebenfalls signifikant ab von 82 % (177/216) auf 45 % (17/38; *p* < 0,001), während der absolute Anteil an Frauen mit jeweils 21–39 Frauen pro Jahr konstant blieb. Es zeigte sich eine signifikante Zunahme im Durchschnittsalter der Patienten im Zeitraum von 2006 bis 2016 von 67,9 Jahren auf 70,7 Jahre (*p* < 0,001).

**Figure 2:**
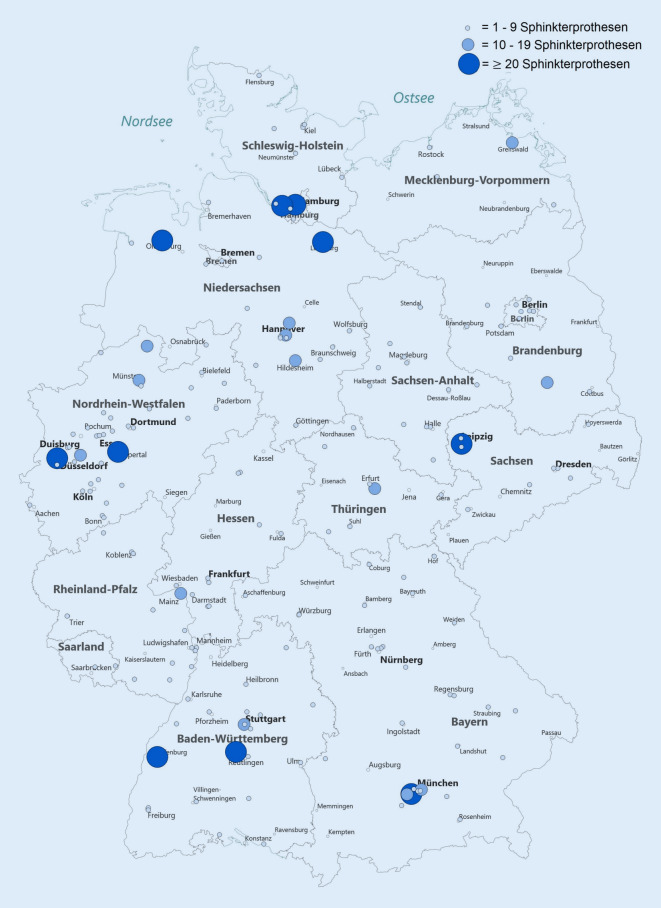

In Deutschland wurden 2016 839 Sphinkterprothesen in 191 urologischen Kliniken implantiert (Tab. 1 und Abb. 2). 13 Sphinkterprothesen wurden jeweils durch zwei chirurgische und zwei gynäkologische Kliniken implantiert. 168 (88 %) der urologischen Kliniken implantierten 1–9 Sphinkterprothesen, 23 (12 %) Kliniken implantierten ≥ 10 Sphinkterprothesen. Kliniken mit geringer Fallzahl (1-9) implantierten 48 % (404/839) der Sphinkter. Kliniken mit hoher Fallzahl (≥10) implantierten 52 % (435/839) der Sphinkter. Die 10 Top-Kliniken (≥20 Sphinkter) implantierten 34 % (283/839) aller Sphinkter. Kliniken mit einer Fallzahl ≥ 10 Sphinkterprothesen/Jahr sind häufiger Universitätskliniken (35 % vs. 11 %, *p* = 0,002). Es gibt keinen signifikanten Zusammenhang zur Größe der urologischen Klinik (*p* = 0,6), der Stadtgröße (*p* = 0,1) und der jährlichen RP-Fallzahl (*p* = 0,1; Tab. 1).

**Table 1:** 

Variable		Gesamt (*n* = 191)	Implantationen 2016		*p*-Wert
			1–9 (*n* = 168)	≥10 (*n* = 23)	
Gesamtzahl der implantierten Sphinkterprothesen		839	404	435	n/a
Bettenzahl des Klinikums	≤ 100	4 (2 %)	2 (1 %)	2 (9 %)	0,058
	101–300	36 (19 %)	32 (19 %)	4 (17 %)	
	301–800	103 (54 %)	94 (56 %)	9 (39 %)	
	> 800	48 (25 %)	40 (24 %)	8 (35 %)	
Bettenzahl der Urologischen Abteilung (12 fehlend)	≤ 10	6 (3 %)	6 (4 %)	0 (0 %)	0,6
	11–20	39 (22 %)	35 (22 %)	4 (18 %)	
	21–50	87 (48 %)	74 (47 %)	13 (59 %)	
	> 50	47 (27 %)	42 (27 %)	5 (23 %)	
Einwohnerzahl der Stadt	< 20.000	19 (10 %)	16 (10 %)	3 (13 %)	0,1
	20.–100.000	76 (40 %)	68 (40 %)	8 (35 %)	
	100.–500.000	60 (31 %)	56 (33 %)	4 (17 %)	
	> 500.000	37 (19 %)	28 (17 %)	8 (35 %)	
Universitätsklinikum	Ja	27 (14 %)	19 (11 %)	8 (35 %)	*0,002*
	Nein	165 (86 %)	149 (89 %)	15 (65 %)	
Prostatakarzinomzentrum (12 fehlend)	Ja	70 (39 %)	58 (37 %)	12 (57 %)	0,1
	Nein	110 (61 %)	99 (63 %)	10 (43 %)	
RP-Fallzahl		81,7 ± 115,5 48,0 (0–979)	75,8 ± 108,6 42,5 (0–979)	124,6 ± 153,0 59,0 (2–564)	0,1

*RP* radikale Prostatektomie

### Penisprothetik

**Figure 3:**
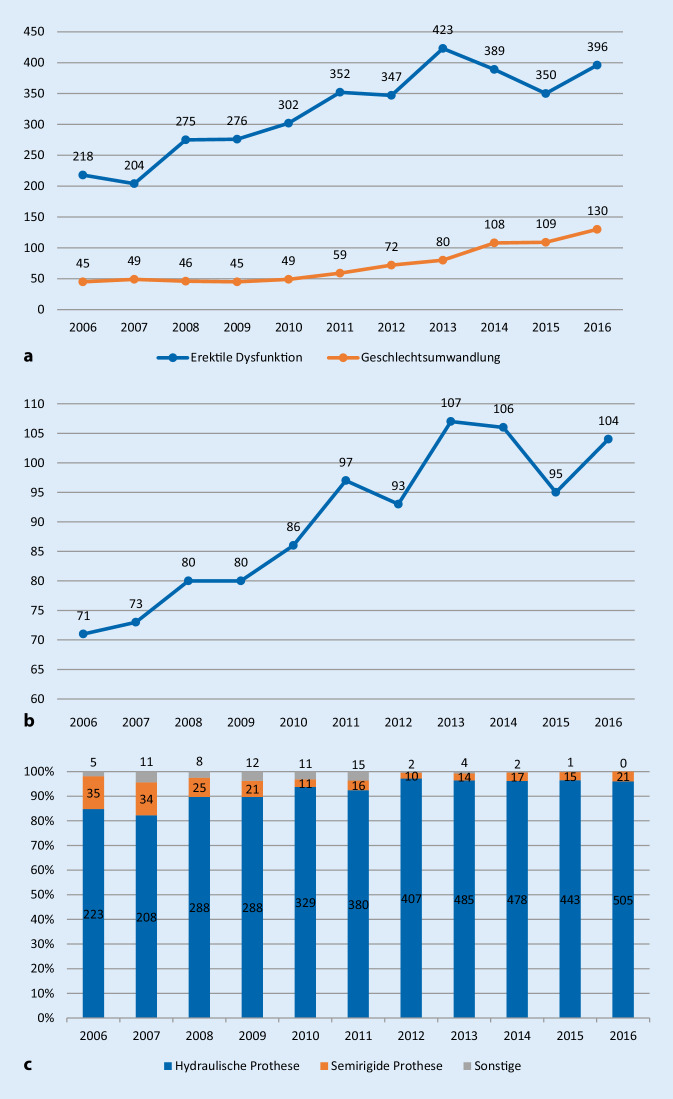

Von 2006 bis 2013 zeigt sich ein kontinuierlicher Anstieg der implantierten Penisprothesen von 263 auf 503 (+33/Jahr, *p* < 0,001), sowie ein Anstieg der implantierenden Kliniken von 71 auf 107 (+5/Jahr, *p* < 0,001). Ab 2013 bis 2016 stagnierten die Fallzahl (*p* = 0,9) und die Zahl der implantierenden Kliniken (*p* = 0,5). Der Anteil hydraulischer Prothesen nahm von 2006 bis 2016 von 85 % (223/263) auf 96 % (505/526) zu (*p* = 0,02), während der Anteil der semirigiden Prothesen von 13 % (35/263) auf 4 % (21/526) sank (*p* = 0,002). Weiterhin nahm der Anteil implantierter Penisprothesen im Rahmen von Geschlechtsumwandlungen von 17 % (45/263) im Jahr 2006 auf 25 % (130/526) im Jahr 2016 zu (*p* = 0,03; Abb. 3). Es zeigte sich keine signifikante Entwicklung im Durchschnittsalter der Patienten von 2006 bis 2016 (*p* = 0,9).

**Figure 4:**
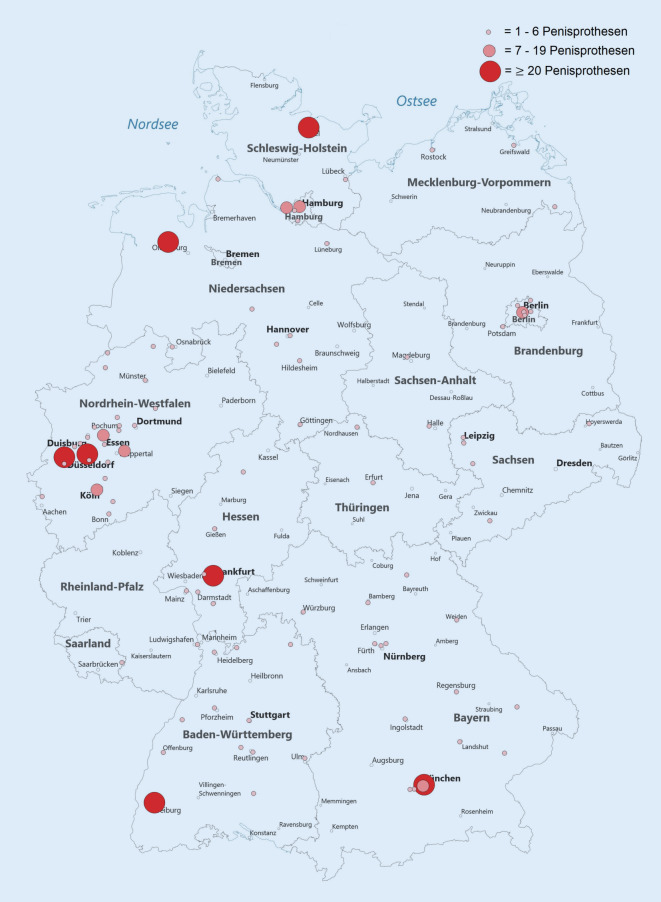

2016 wurden 448 Penisprothesen in 97 urologischen Kliniken in Deutschland implantiert (Tab. 2 und Abb. 4). 45 Penisprothesen wurden durch 6 chirurgische Kliniken implantiert. 83 (86 %) der urologischen Kliniken implantierten 1–6 Penisprothesen, 7 (7 %) Kliniken implantierten 7–20 Prothesen, 6 (6 %) Kliniken 21–32 Prothesen und 1 (1 %) Klinik 71 Penisprothesen. Kliniken mit geringer Fallzahl (≤6) implantierten 144/448 (32 %) Penisprothesen und Kliniken mit hoher Fallzahl (>6) implantierten 304/448 (68 %) Penisprothesen. Die 7 Top-Kliniken (≥20 Prothesen/Jahr) implantierten 232/448 (52 %) der Prothesen. Kliniken mit einer Fallzahl > 6 Penisprothesen/Jahr sind häufiger Universitätskliniken (50 % vs. 19 %, *p* = 0,01), liegen häufiger in Großstädten (64 % vs. 16 %, *p* = 0,001) und haben eine höhere Fallzahl an Patienten mit Störung der Geschlechtsidentität (42,2 ± 109,5 vs. 1,7 ± 12,6; *p* < 0,001). Es gibt keinen signifikanten Zusammenhang zur Größe der urologischen Klinik (*p* = 0,5) und jährlichen RP-Fallzahl (*p* = 0,7; Tab. 2).

**Table 2:** 

Variable		Gesamt (*n* = 97)	Implantationen 2016		*p*-Wert
			1–6 (*n* = 83)	>6 (*n* = 14)	
Zahl der implantierten Penisprothesen		448	144	304	n/a
Bettenzahl des Klinikum (3 fehlend)	≤ 100	2 (2 %)	1 (1 %)	1 (8 %)	0,09
	101–300	14 (15 %)	13 (16 %)	1 (8 %)	
	301–800	45 (48 %)	42 (51 %)	3 (25 %)	
	> 800	33 (35 %)	26 (32 %)	7 (59 %)	
Bettenzahl der Urologischen Abteilung (5 fehlend)	≤ 10	3 (3 %)	3 (4 %)	0 (0 %)	0,5
	11–20	16 (18 %)	15 (19 %)	1 (8 %)	
	21–50	49 (53 %)	43 (53 %)	6 (50 %)	
	> 50	24 (26 %)	19 (24 %)	5 (42 %)	
Einwohnerzahl der Stadt	< 20.000	10 (10 %)	10 (12 %)	0 (0 %)	*0,001*
	20.–100.000	27 (28 %)	25 (30 %)	2 (14 %)	
	100.–500.000	38 (39 %)	35 (42 %)	3 (22 %)	
	> 500.000	22 (23 %)	13 (16 %)	9 (64 %)	
Universitätsklinikum	Ja	23 (24 %)	16 (19 %)	7 (50 %)	*0,01*
	Nein	74 (76 %)	67 (81 %)	7 (50 %)	
Prostatakarzinomzentrum (5 fehlend)	Ja	32 (35 %)	28 (35 %)	4 (33 %)	0,9
	Nein	60 (65 %)	52 (65 %)	8 (67 %)	
RP-Fallzahl		110,6 ± 151,4 55,5 (0–979)	105,0 ± 148,7 55,5 (0–979)	143,3 ± 168,2 74,5 (2–486)	0,7
Fallzahl mit Störung der Geschlechtsidentität (ICD F64)		7,6 ± 44,3 0 (0–415)	1,7 ± 12,6 0 (0–113)	42,2 ± 109,5 0,5 (0–415)	*<0,001*

*RP* radikale Prostatektomie

**Figure 5:**
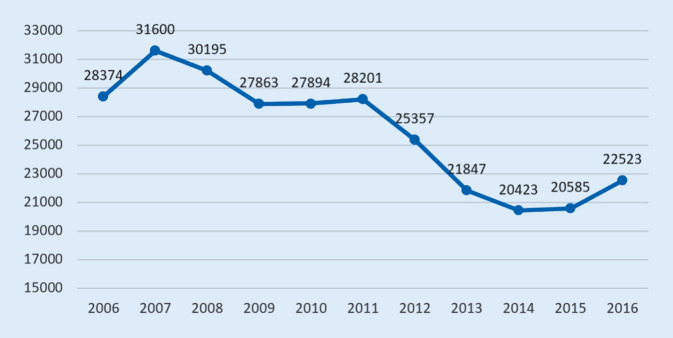

Zur besseren Einordnung eines möglichen Versorgungsdefizits zeigt Abb. 5 die jährlichen Fallzahlen der RP in Deutschland von 2006 bis 2016.

## Diskussion

Bei der Implantation von Sphinkterprothesen zeigte sich von 2006 bis 2012 ein kontinuierlicher Anstieg der Gesamtfallzahl (+76/Jahr, *p* < 0,001) und der implantierenden Kliniken (+15/Jahr, *p* < 0,001). Seit 2012 nahm die Fallzahl (−40/Jahr, *p* = 0,02) und die Klinikzahl (−2/Jahr, *p* = 0,03) ab. Die 10 Top-Kliniken (≥20 Sphinkter) implantierten ein Drittel aller 839 Sphinkter. Es zeigte sich ein deutlicher Abfall des Einsatzes von Sphinktern mit Blasenmanschetten von 29 % auf 4 % aufgrund des geringeren Einsatzes bei Männern bei gleichbleibenden Implantationszahlen bei Frauen.

Bei der Implantation von Penisprothesen zeigt sich ebenfalls ein initialer Anstieg der Fallzahlen (+33/Jahr, *p* < 0,001) und implantierenden Kliniken 107 (+5/Jahr, *p* < 0,001) bis 2013. Ab 2013 stagnieren die Fallzahlen und die Zahl der Kliniken. Lediglich der Anteil implantierter Penisprothesen im Rahmen von Geschlechtsumwandlungen nahm kontinuierlich von 2006 bis 2016 zu (*p* = 0,03). Die 7 Top-Kliniken (≥20 Prothesen/Jahr) implantierten die Hälfte aller 448 Prothesen. Es gab bei der Fallzahl der Sphinkterprothesen und der Penisprothesen keinen Zusammenhang mit der Fallzahl der radikalen Prostatektomien der jeweiligen Klinik (*p* = 0,1 bzw. *p* = 0,7).

Stressinkontinenz und erektile Dysfunktion nach RP stellen die häufigsten Indikationen für die Implantation von Sphinkter- und Penisprothese dar. Laut der großen deutschen Versorgungsforschungsstudie HAROW (Hormontherapie, Active Surveillance, Radiotherapie, Operation und Watchful Waiting) erhalten 56,6 % der Patienten mit lokal begrenzten Prostatakarzinom eine RP, 16,4 % eine Radiatio und 27 % ein konservatives Vorgehen [11, 12]. In der aktuellen Follow-up-Studie dieser Patienten nach RP betrug die Inkontinenzrate 15 %. Die Rate der erektilen Dysfunktion betrug insgesamt 86 % und bei der Subkategorie der präoperativ potenten Patienten nach nerverhaltender Operation 58 % [13]. Einschränkungen des funktionellen Outcomes beeinflussen nicht nur die Lebensqualität negativ, sondern führen auch zu einem stärkeren Bedauern der Entscheidung zur Operation [14, 15]. Entsprechend kann die Therapie der Inkontinenz und erektilen Dysfunktion entscheidend für die Lebensqualität der Patienten sein. In der Analyse der Follow-up-Patienten mit funktionellen Einschränkungen der HAROW-Studie zeigte sich, dass nur 25 % der inkontinenten Patienten eine operative Therapie erhielten und nur 51 % der Patienten mit erektiler Dysfunktion und Interesse an Sex, Hilfsmittel nutzen oder zumindest einmal ausprobiert haben [9]. In dieser Studie zeigte sich bereits ein Versorgungsdefizit, da die Hälfte der inkontinenten Patienten ohne Operation eine deutlich eingeschränkte Lebensqualität aufgrund ihrer Inkontinenz aufwies. Bei Patienten mit erektiler Dysfunktion und Interesse an Sex, welche noch nie Hilfsmittel zur Verbesserung ihrer erektilen Funktion genutzt hatten, berichten ein Drittel eine deutlich eingeschränkte Lebensqualität aufgrund ihrer Einschränkung. Offen bleiben die genauen Gründe für dieses Versorgungsdefizit. Einige Studien zeigen ein Defizit in Wahrnehmung und Kommunikation bezüglich Inkontinenz und erektiler Dysfunktion zwischen Arzt und Patient auf [16–18]. Oft ist das Internet erster Anlaufpunkt für Patienten zur Informationssuche und dem Austausch mit anderen Betroffenen [19–22].

Betrachtet man in Abb. 5 die Anzahl von 22.500 RP im Jahr 2016 und leitet hier die oben genannte Raten an Inkontinenz und erektiler Dysfunktion ab, kann man von etwa 3300 Patienten mit Inkontinenz und 19.000 Patienten mit erektiler Dysfunktion ausgehen. Somit ergibt sich ein relevanter Bedarf an Sphinkter- und Penisprothesenimplantationen, auch wenn es bei beiden funktionellen Einschränkungen zahlreiche operative bzw. medikamentöse Therapiealternativen gibt. Unsere Studie zeigt auf, dass die Zahl der Sphinkter- und Penisprothesenimplantationen nicht mit der Zahl der RP der einzelnen Kliniken korreliert (*p* = 0,1 bzw. *p* = 0,7). Dies ist ein Hinweis darauf, dass viele Klinik nicht die funktionellen Folgen ihrer RP selbst therapieren.

Auch andere Arbeiten ergaben bereits Hinweise auf ein Versorgungsdefizit bei der Behandlung funktioneller Einschränkungen nach RP. Eine Auswertung der SEER(Surveillance, Epidemiology, and End Results Program)-Datenbank in den USA von 16.348 Männern nach RP zeigte, dass nur 6 % der Patienten eine Inkontinenzoperation erhielten. Bei einer deutlich höheren Inkontinenzrate nach RP stellt dies einen Hinweis auf eine Unterversorgung dar [23]. Eine weitere Auswertung der SEER-Datenbank zeigte, dass 2,3 % der Patienten nach RP eine Penisprothese erhalten haben [24]. Eine deutsche Studie zeigte nur eine Nutzung von 0,3 % [25] und das Follow-up von 936 Patienten nach RP im Rahmen der HAROW-Studie erbrachte keinen einzigen Patienten mit Penisprothese [9].

Die einzige konstante Steigerung von 2006 bis 2016 zeigt sich im Einsatz der Penisprothese bei Geschlechtsumwandlungen. Hier verdreifachte sich die Zahl von 38 Implantationen 2006 auf 120 im Jahr 2016. Dies spiegelt die Zunahme an Geschlechtsumwandlungen in Deutschland wider [26]. Bei der Implantation von Penisprothesen bei erektiler Dysfunktion zeigt sich ab 2013 eine Stagnation, bei Sphinkterporthesenimplantationen ab 2012 sogar ein leichter Abfall. Für beide Entwicklungen kann zum einen die deutliche Abnahme von radikalen Prostatektomien ab 2011 verantwortlich sein [27]. Zum anderen konkurriert auch das ATOMS („adjustable transobturator male system“) als operative Alternative zur Sphinkterprothese [28].

Bei der Implantation von Sphinkter- und Penisprothesen zeigt sich eine für deutsche Verhältnisse recht ausgeprägte Zentrumsbildung. Am stärksten ist diese bei Penisprothesen ausgebildet. Hier implantieren die 7 Top-Kliniken (≥20 Prothesen/Jahr, 7 % aller Kliniken) die Hälfte aller Penisprothesen in Deutschland. Penisprothesen werden überwiegend in Großstädten und Universitätskliniken implantiert. Bei Sphinkterprothesen implantierten die 10 Top-Kliniken (≥20 Prothesen/Jahr, 5 % aller Kliniken) ein Drittel der Sphinkterprothesen in Deutschland. Aufgrund der relevanten Komplikations- und Revisionsraten bei der urologischen Endoprothetik [4, 7] ist eine Zentrumsbildung mit entsprechend höherer operativer Erfahrung zu befürworten [5, 8]. Eine große Kohortenstudien in den USA mit 65.602 Patienten zeigte, dass selbst mit einer Erfahrung von über 200 Sphinkterprothesenimplantationen eine weitere Qualitätssteigerung möglich ist [5], wobei ein Großteil der Patienten von Urologen operiert wird, die diese Erfahrung nie erreichen [29]. Trotz der Zentrumsbildung in Deutschland wiesen 2016 88 % der Kliniken mit Sphinkterprothesenimplantation ≤ 9 Fälle/Jahr bzw. 86 % der Klinik mit Penisprothesenimplantation ≤ 6 Fälle/Jahr auf.

In der Fallzahlentwicklung des Einsatzes der Sphinkterprothesen zeigt sich eine wesentliche Entwicklung bei Manschetten im Bereich des Blasenhalses. Hier nahm der Einsatz mit einem initialen Anteil von 29 % auf 4 % deutlich ab. In der Subgruppenanalyse zeigte sich ein möglicher Grund in der deutlichen Abnahme bei männlichen Patienten von 82 % auf 45 %, während der absolute Anteil der Frauen gleichblieb (2006 39 Frauen und 177 Männer, 2016 21 Frauen und 17 Männer). Auch bei Penisprothesen zeigt sich eine Entwicklung des Einsatzes der beiden Typen mit einem zunehmenden Einsatz der hydraulischen Prothese, welche deutlich komfortabler für die Patienten ist [30].

Dies ist die erste Studie zur Versorgungswirklichkeit der Implantation von Sphinkter- und Penisprothesen in Deutschland. Die Nutzung von DRG-Abrechnungsdaten und der Qualitätsberichte ermöglicht eine sehr genaue Darstellung der Versorgungssituation. Die Fallzahlangaben der Qualitätsberichte liegen leicht unter den Angaben der DRG-Daten, da bei den Qualitätsberichten Fallzahlen von 1–3 als 1 kodiert werden. Somit war eine exakte Differenzierung der niedrigen Fallzahlen aufgrund der Zahl an OPS-Subkategorien bei Sphinkterprothesen (3 Kategorien) nur bis mindestens 9 und bei Penisprothesen (2 Kategorien) bis mindestens 6 möglich. Die DRG-Datenbank kann nicht zwischen Fachbereichen differenzieren, sodass hier auch nicht-urologische Kliniken eingeschlossen sind. In der Analyse der Qualitätsberichte zeigt sich aber dieser Anteil (1/98 Kliniken mit Penisprothesenimplantation und 3/195 Kliniken mit Sphinkterprothesenimplantation) als sehr gering. Weiterhin erfasst die DRG-Datenbank nicht Implantationen, welche außerhalb des DRG-Systems abgerechnet wurden. In beiden Datenbanken fehlen klinische Daten, sodass weder die Indikation noch die Versorgungsqualität der Implantationen näher charakterisiert werden kann. Aufgrund der anonymen Datenquellen lässt sich beispielsweise auch nicht analysieren, ob Revisionseingriffe bei den eigenen Patienten oder möglicherweise bei zugewiesenen, extern implantierten Patienten erfolgten. Die Darstellung der Fallzahl einer Klinik im Jahr 2016 stellt überdies nur eine Momentaufnahme dar und kann in davor und danach liegenden Jahren variieren.

## Fazit für die Praxis

- Der Versorgungsstand der urologischen Endoprothetik in Deutschland zeigt eine deutliche Zentrenbildung, aber auch eine große Zahl von Kliniken mit geringer Fallzahl.
- Da bei beiden Prothesen ein relevantes Komplikations- und Revisionsrisiko besteht, ist die operative Erfahrung entscheidend bei der Implantation.
- Seit 2012/2013 zeigt sich eine Stagnation der Fallzahlen von Penis- und Sphinkterprothesenimplantationen, die in Zusammenschau mit den Prostatektomiefallzahlen eine Unterversorgung vermuten lässt.

## Supplementary Information




