# Supplementary material for: Health care reality of urological endoprosthetics in Germany from 2006 to 2016
Source: Urologe A. 2021 Jan 22;60(3):351–60. [Article in German] doi: 10.1007/s00120-021-01444-5 (PMC7979589; doi:10.1007/s00120-021-01444-5)
Supplement: Supplementary file 1 [file 120_2021_1444_MOESM1_ESM.docx]

| Klinik | Abteilung | Ort | Fallzahl Sphinkterprothesen-implantation |
| --- | --- | --- | --- |
| Klinikum der Universität München | Urologische Klinik und Poliklinik | München | 38 |
| Universitätsklinikum Hamburg-Eppendorf | Klinik und Poliklinik für Urologie | Hamburg | 37 |
| Ortenau Klinikum Offenburg-Gengenbach Standort Ebertplatz | Urologie und Kinderurologie | Offenburg | 35 |
| Asklepios Westklinikum Hamburg | Abteilung für Urologie | Hamburg | 32 |
| HELIOS Klinikum Schwelm | Klinik für Urologie und Kinderurologie | Schwelm | 26 |
| Städtisches Klinikum Lüneburg | Klinik für Urologie | Lüneburg | 25 |
| Universitätsklinikum Leipzig AöR | Klinik und Poliklinik für Urologie | Leipzig | 24 |
| Ammerland-Klinik Westerstede | Klinik für Urologie und Kinderurologie | Westerstede | 22 |
| Krankenhaus St. Franziskus | Klinik für Urologie | Mönchengladbach | 22 |
| Universitätsklinikum Tübingen | Universitätsklinik für Urologie | Tübingen | 22 |
| KRH Klinikum Großburgwedel | Urologie | Burgwedel | 15 |
| Katholisches Krankenhaus "St. Johann Nepomuk" | Klinik für Urologie und Kinderurologie | Erfurt | 14 |
| Universitätsklinikum Münster | Klinik für Urologie und Kinderurologie | Münster | 13 |
| Diakonie-Klinikum Stuttgart | Urologische Klinik | Stuttgart | 13 |
| St. Elisabeth-Krankenhaus | Urologie | Ibbenbüren | 12 |
| Universitätsmedizin der Johannes Gutenberg-Universität Mainz | Klinik und Poliklinik für Urologie und Kinderurologie | Mainz | 12 |
| Chirurgische Klinik München-Bogenhausen | Urologie | München | 12 |
| Universitätsmedizin Greifswald | Klinik und Poliklinik für Urologie | Greifswald | 11 |
| Medizinische Hochschule Hannover | Klinik für Urologie und Urologische Onkologie | Hannover | 10 |
| St. Bernward Krankenhaus | Urologische Klinik | Hildesheim | 10 |
| Paracelsus Klinik Düsseldorf Golzheim | Urologie | Düsseldorf | 10 |
| Urologische Klinik München - Planegg | Urologie | Planegg | 10 |
| Evangelisches Krankenhaus Luckau | Klinik für Urologie | Luckau | 10 |
| Universitätsklinikum Köln | Klinik für Urologie, Uro-Onkologie, spezielle urologische und roboter-assistierte Chirurgie | Köln | 9 |
| Prosper-Hospital | Klinik für Urologie & Zentrum für Minimalinvasive und Robotische Chirurgie in der Urologie | Recklinghausen | 9 |
| Schwarzwald-Baar Klinikum Villingen-Schwenningen | Klinik für Urologie und Kinderurologie | Villingen-Schwenningen | 9 |
| Universitätsklinikum Freiburg | Klinik für Urologie | Freiburg | 9 |
| Caritas-Krankenhaus St. Josef | Klinik für Urologie | Regensburg | 9 |
| Krankenhaus der Barmherzigen Brüder Trier | Urologie und Kinderurologie | Trier | 8 |
| Klinikum Worms | Urologische Klinik - Klinik für Urologie und Kinderurologie | Worms | 8 |
| Vivantes Humboldt-Klinikum | Klinik für Urologie | Berlin | 8 |
| Johanniter-Krankenhaus Genthin-Stendal | Urologie | Stendal | 8 |
| Universitätsklinikum Bonn | Klinik und Poliklinik für Urologie und Kinderurologie | Bonn | 7 |
| St. Antonius-Hospital Gronau | Klinik für Urologie, Kinderurologie und Urologische Onkologie | Gronau | 7 |
| Universitätsklinikum Mannheim | Urologische Universitätsklinik | Mannheim | 7 |
| KMG Klinikum Güstrow | Klinik für Urologie | Güstrow | 7 |
| Universitätsklinikum Halle (Saale) | Universitätsklinik und Poliklinik für Urologie | Halle (Saale) | 7 |
| Krankenhaus Martha-Maria Halle-Dölau | Klinik für Urologie, Kinderurologie und urologische Onkologie | Halle (Saale) | 7 |
| Eichsfeld Klinikum | Urologie Haus Reifenstein | Kleinbartloff OT Reifenstein | 7 |
| Uinversitätsklinikum Schleswig-Holstein, Campus Kiel | Klinik für Urologie und Kinderurologie | Kiel | 6 |
| GFO Kliniken Troisdorf, Betriebsstätte St. Josef Troisdorf | Urologie | Troisdorf | 6 |
| Klinikum der Stadt Ludwigshafen am Rhein | Urologische Klinik | Ludwigshafen | 6 |
| Loretto-Krankenhaus (RkK) Freiburg | Urologie und urologische Onkologie | Freiburg | 6 |
| Klinikum Magdeburg | Klinik für Urologie und Kinderurologie | Magdeburg | 6 |
| Städtisches Krankenhaus Kiel | Klinik für Urologie | Kiel | 5 |
| Universitätsklinikum Essen | Klinik für Urologie | Essen | 5 |
| medius Klinik Ostfildern-Ruit | Klinik für Urologie | Ostfildern | 5 |
| St. Anna-Virngrund-Klinik | Urologie | Ellwangen | 5 |
| Klinikum Mittelbaden Baden-Baden Balg | Klinik für Urologie | Baden-Baden | 5 |
| Kliniken Nordoberpfalz - Klinikum Weiden | Klinik für Urologie, Andrologie und Kinderurologie | Weiden i. d. OPf. | 5 |
| Klinikum Kulmbach | Urologie | Kulmbach | 5 |
| Universitätsklinikum des Saarlandes | Klinik für Urologie und Kinderurologie | Homburg | 5 |
| Asklepios Klinik Pasewalk | Klinik für Urologie | Pasewalk | 5 |
| Dessau-Roßlau | Klinik für Urologie, Kinderurologie und urologische Onkologie | Dessau-Roßlau | 5 |
| SRH Wald-Klinikum Gera | Klinik für Urologie und Kinderurologie | Gera | 5 |
| Asklepios Klinikum Harburg | Urologie | Hamburg | 4 |
| Uniklinik RWTH Aachen | Klinik für Urologie | Aachen | 4 |
| Alice-Hospital | Urologie | Darmstadt | 4 |
| Universitätsklinikum Frankfurt | Urologie | Frankfurt am Main | 4 |
| Krankenhaus Maria Hilf | Urologie | Bad Neuenahr-Ahrweiler | 4 |
| Vinzentius-Krankenhaus Landau | Klinik für Urologie und Kinderurologie | Landau | 4 |
| Klinikum am Steinenberg | Urologische Klinik | Reutlingen | 4 |
| Klinikum Ingolstadt | Urologie | Ingolstadt | 4 |
| Evangelisches Krankenhaus Königin Elisabeth Herzberge | Urologie | Berlin | 4 |
| St. Elisabeth-Krankenhaus | Urologie | Leipzig | 4 |
| Universitätsmedizin Göttingen | Klinik für Urologie | Göttingen | 3 |
| Evangelisches Klinikum Niederrhein | Klinik für Urologie | Oberhausen | 3 |
| Klinikum Aschaffenburg-Alzenau | Klinik für Urologie und Kinderurologie | Aschaffenburg | 3 |
| Universitätsmedizin Rostock | Urologische Klinik und Poliklinik | Rostock | 3 |
| Helios Kliniken Mittelweser | Urologie | Nienburg | 2 |
| Städtische Kliniken Mönchengladbach | Klinik für Urologie und Kinderurologie | Mönchengladbach | 2 |
| Heilig Geist-Krankenhaus | Klinik für Urologie | Köln | 2 |
| St. Josef-Krankenhaus, Hamm Bockum-Hövel | Klinik für Urologie, Kinderurologie und Uro-Gynäkologie | Hamm-Bockum-Hovel | 2 |
| Agaplesion Markus Krankenhaus | Urologische Klinik | Frankfurt am Main | 2 |
| Krankenhaus Hetzelstift | Klinik für Urologie, Kinderurologie und urologische Onkologie | Neustadt an der Weinstraße | 2 |
| Sankt Vincentius Krankenhaus | Klinik für Urologie | Speyer | 2 |
| Caritas-Krankenhaus | Urologie | Bad Mergentheim | 2 |
| Klinikum Starnberg | Urologie | Starnberg | 2 |
| Klinikum St. Elisabeth Straubing | Klinik für Urologie | Straubing | 2 |
| Sana Klinikum Hof | Klinik für Urologie, Kinderurologie, urologische Onkologie und Palliativmedizin | Hof | 2 |
| Klinikum Fürth | Klinik für Urologie und Kinderurologie | Fürth | 2 |
| Missionsärztliche Klinik | Urologie | Würzburg | 2 |
| Charité - Universitätsmedizin Berlin | Klinik für Urologie | Berlin | 2 |
| DRK Krankenhaus Luckenwalde | Urologie | Luckenwalde | 2 |
| Heinrich-Braun-Klinikum, Standort Zwickau | Klinik für Urologie | Zwickau | 2 |
| Kreiskrankenhaus Freiberg | Klinik für Urologie und Kinderurologie | Freiberg | 2 |
| Universitätsklinikum Dresden | Klinik und Poliklinik für Urologie | Dresden | 2 |
| HELIOS Klinikum Erfurt | Urologie | Erfurt | 2 |
| Diakonissenkrankenhaus Flensburg | Urologische Klinik | Flensburg | 1 |
| Regio Kliniken - Klinikum Wedel | Klinik für Urologie | Wedel | 1 |
| imland Klinik Rendsburg | Klinik für Urologie | Rendsburg | 1 |
| FEK - Friedrich-Ebert-Krankenhaus Neumünster | Klinik für Urologie | Neumünster | 1 |
| HELIOS Klinik Kiel | Urologie | Kiel | 1 |
| Uuniversitätsklinikum Schleswig-Holstein, Campus Lübeck | Klinik für Urologie | Lübeck | 1 |
| Asklepios Klinik Altona | Urologie | Hamburg | 1 |
| Josef-Hospital Delmenhorst Krankenhaus | Klinik für Urologie und Kinderurologie | Delmenhorst | 1 |
| Städtisches Klinikum Braunschweig | Klinik für Urologie und Uroonkologie | Braunschweig | 1 |
| HELIOS Klinikum Salzgitter | Urologie | Salzgitter | 1 |
| Klinikum Wolfsburg | Urologische Klinik | Wolfsburg | 1 |
| Gesundheitseinrichtungen Hameln-Pyrmont - Sana Klinikum | Klinik für Urologie | Hameln | 1 |
| DIAKOVERE Friederikenstift | Urologie | Hannover | 1 |
| Vinzenzkrankenhaus Hannover | Urologie | Hannover | 1 |
| KRH Klinikum Siloah | Urologie | Hannover | 1 |
| AMEOS Klinikum Seepark Geestland | Klinik für Urologie und Kinderurologie | Geestland | 1 |
| Agaplesion Diakonieklinikum Rotenburg | Klinik für Urologie und Kinderurologie | Rotenburg (Wümme) | 1 |
| Borromäus Hospital Leer | Urologie und Kinderurologie | Leer | 1 |
| Klinikum Bremen-Mitte | Urologische Klinik | Bremen | 1 |
| Alfried Krupp Krankenhaus Steele | Klinik für Urologie und urologische Onkologie | Essen | 1 |
| Kliniken Essen-Mitte Evang. Huyssens-Stiftung | Klinik für Urologie, Kinderurologie und Urologische Onkologie | Essen | 1 |
| HELIOS Klinikum Krefeld | Klinik für Urologie und Kinderurologie | Krefeld | 1 |
| Alexianer Krefeld - Krankenhaus Maria-Hilf | Klinik für Urologie und Kinderurologie | Krefeld | 1 |
| Städtisches Klinikum Solingen | Klinik für Urologie und Kinderurologie | Solingen | 1 |
| St. Josef Krankenhaus Moers - Betriebsstätte St. Josef Krankenhaus Moers | Urologie | Moers | 1 |
| Städtische Kliniken Neuss - Lukaskrankenhaus | Urologische Klinik | Neuss | 1 |
| Waldkrankenhaus Bonn | Urologie | Bonn | 1 |
| Klinikum Leverkusen | Klinik für Urologie | Leverkusen | 1 |
| St.-Katharinen-Hospital | Urologie | Frechen | 1 |
| St. Josef Krankenhaus | Urologie | Engelskirchen | 1 |
| Herz-Jesu-Krankenhaus Hiltrup | Klinik für Urologie, Onkologische Urologie und Neuro-Urologie | Münster | 1 |
| Marien-Hospital Marl | Klinik für Urologie | Marl | 1 |
| Evangelisches Krankenhaus Bielefeld - Standort Johannesstift | Klinik für Urologie | Bielefeld | 1 |
| Klinikum Gütersloh | Klinik für Urologie | Gütersloh | 1 |
| Klinikum Lippe Detmold | Klinik für Urologie | Detmold | 1 |
| Krankenhaus Lübbecke-Rahden, Betriebsstelle Lübbecke | Klinik für Urologie, Kinderurologie und Operative Uro-Onkologie | Lübbecke | 1 |
| Brüderkrankenhaus St. Josef Paderborn | Klinik für Urologie und Kinderurologie | Paderborn | 1 |
| Augusta-Kranken-Anstalt | Urologie | Bochum | 1 |
| Klinikum Dortmund Nord | Urologische Klinik | Dortmund | 1 |
| Marien-Hospital | Urologie | Erwitte | 1 |
| Knappschaftskrankenhaus Dortmund, Klinikum Westfalen | Klinik für Urologie und Kinderurologie | Dortmund | 1 |
| Klinikum Darmstadt | Urologische Klinik | Darmstadt | 1 |
| HELIOS Dr. Horst-Schmidt-Kliniken | Klinik für Urologie und Kinderurologie | Wiesbaden | 1 |
| Krankenhaus Eichhof Lauterbach | Urologie | Lauterbach | 1 |
| Klinikum Fulda | Urologie | Fulda | 1 |
| Werner Wicker Klinik - Orthopädisches Schwerpunktklinikum Werner Wicker | Abteilung für Neuro-Urologie | Bad Wildungen | 1 |
| GPR Klinikum | Klinik für Urologie, Kinderurologie und onkologische Urologie | Rüsselsheim | 1 |
| Asklepios Stadtklinik Bad Wildungen | Abteilung für Urologie | Bad Wildungen | 1 |
| Gemeinschaftsklinikum Mittelrhein, Kemperhof | Klinik für Urologie und Kinderurologie | Koblenz | 1 |
| Diakonie Krankenhaus Kreuznacher Diakonie | Urologie | Bad Kreuznach | 1 |
| Bundeswehr Zentralkrankenhaus Koblenz | Klinik für Urologie | Koblenz | 1 |
| Städtisches Krankenhaus Pirmasens | Urologie und Kinderurologie | Pirmasens | 1 |
| Klinikum Ludwigsburg | Klinik für Urologie | Ludwigsburg | 1 |
| Rems-Murr-Klinikum Winnenden | Urologie | Winnenden | 1 |
| SLK-Kliniken Heilbronn - Klinikum am Gesundbrunnen | Klinik für Urologie und Kinderurologie | Heilbronn | 1 |
| Klinikum Stuttgart - Katharinenhospital | Klinik für Urologie und Transplantationschirurgie | Stuttgart | 1 |
| Städtisches Klinikum Karlsruhe | Urologische Klinik | Karlsruhe | 1 |
| Diakonissenkrankenhaus Mannheim | Klinik für Urologie | Mannheim | 1 |
| Siloah St. Trudpert Klinikum | Klinik für Urologie | Pforzheim | 1 |
| Hegau-Bodensee-Klinikum Singen | Klinik für Urologie und Kinderurologie | Singen | 1 |
| Klinikum Friedrichshafen | Klinik für Urologie, Kinderurologie und urologische Onkologie | Friedrichshafen | 1 |
| SRH Krankenhaus Sigmaringen | Urologie | Sigmaringen | 1 |
| Bundeswehrkrankenhaus Ulm | Abteilung für Urologie | Ulm | 1 |
| Barmherzige Brüder Krankenhaus München | Urologie | München | 1 |
| RoMed Klinikum Rosenheim | Urologie | Rosenheim | 1 |
| Kreisklinik Ebersberg | Urologie Hauptabteilung | Ebersberg | 1 |
| Klinikum rechts der Isar der Technischen Universität München | Klinik und Poliklinik für Urologie | München | 1 |
| Isar Kliniken | Urologie | München | 1 |
| Klinikum Landshut | Klinik für Urologie | Landshut | 1 |
| Donau Isar Klinikum Deggendorf | Klinik für Urologie und Kinderurologie | Deggendorf | 1 |
| Rottal-Inn Kliniken | Urologie | Eggenfelden | 1 |
| Krankehaus Barmherzige Brüder Regensburg | Klinik für Urologie | Regensburg | 1 |
| Klinikum Neumarkt | Urologische Klinik | Neumarkt | 1 |
| Klinikum Bamberg - Betriebsstätte am Bruderwald | Klinik für Urologie und Kinderurologie | Bamberg | 1 |
| Klinik Hohe Warte | Urologie | Bayreuth | 1 |
| Klinikum Coburg | Klinik für Urologie und Kinderurologie | Coburg | 1 |
| Klinikum Nürnberg Nord | Klinik für Urologie | Nürnberg | 1 |
| St. Theresien-Krankenhaus Nürnberg | Urologie | Nürnberg | 1 |
| Krankenhaus Martha-Maria Nürnberg | Urologisches Zentrum | Nürnberg | 1 |
| Universitätsklinikum Würzburg | Klinik und Poliklinik für Urologie und Kinderurologie | Würzburg | 1 |
| Günzburg | Urologische Praxis | Günzburg | 1 |
| Knappschaftsklinikum Saar GmbH, Krankenhaus Sulzbach | Urologie | Sulzbach | 1 |
| Helios Klinikum Berlin-Buch | Urologie | Berlin | 1 |
| BG-Unfallklinik - Unfallkrankenhaus Berlin | Klinik für Urologie und Neuro-Urologie | Berlin | 1 |
| Vivantes Auguste-Viktoria-Klinikum | Klinik für Urologie | Berlin | 1 |
| Klinikum Ernst von Bergmann | Klinik für Urologie | Potsdam | 1 |
| Städtisches Klinikum Brandenburg | Klinik für Urologie und Kinderurologie | Brandenburg an der Havel | 1 |
| Havelland Kliniken, Klinik Nauen | Klinik für Urologie | Nauen | 1 |
| Carl-Thiem-Klinikum Cottbus | Urologische Klinik | Cottbus | 1 |
| Klinikum St. Georg | Klinik für Urologie und Andrologie | Leipzig | 1 |
| Lausitzer Seenland Klinikum | Klinik für Urologie, Kinderurologie und onkologische Urologie | Hoyerswerda | 1 |
| HELIOS Klinikum Pirna | Klinik für Urologie und Kinderurologie | Pirna | 1 |
| Städtisches Klinikum Dresden - Standort Friedrichstadt | Klinik für Urologie | Dresden | 1 |
| HELIOS Bördeklinik | Klinik für Urologie | Oschersleben / OT Neindorf | 1 |
| Universitätsklinikum Magdeburg | Universitätsklinik für Urologie und Kinderurologie | Magdeburg | 1 |
| Evangelisches Krankenhaus Paul Gerhardt Stift | Urologie | Luth. Wittenberg | 1 |
| HELIOS Klinik Lutherstadt Eisleben | Urologie | Lutherstadt Eisleben | 1 |
| AMEOS Klinikum Haldensleben | Klinik für Urologie, Kinderurologie und Uroonkologie | Haldensleben | 1 |
| Asklepios Klinik Weißenfels | Klinik für Urologie | Weißenfels | 1 |
| AMEOS Klinikum Halberstadt | Klinik für Urologie und Kinderurologie | Halberstadt | 1 |
| Südharz Klinikum Nordhausen | Klinik für Urologie | Nordhausen | 1 |
| HELIOS Klinikum Meiningen | Klinik für Urologie | Meiningen | 1 |
| SRH Zentralklinikum Suhl | Klinik für Urologie | Suhl | 1 |

**Ergänzende Tabelle 1**: Fallzahl der Sphinkterprothesenimplantationen 2016 in urologischen Kliniken in Deutschland.
