# Supplementary material for: Health care reality of urological endoprosthetics in Germany from 2006 to 2016
Source: Urologe A. 2021 Jan 22;60(3):351–60. [Article in German] doi: 10.1007/s00120-021-01444-5 (PMC7979589; doi:10.1007/s00120-021-01444-5)
Supplement: Supplementary file 2 [file 120_2021_1444_MOESM2_ESM.docx]

| Klinik | Abteilung | Ort | Fallzahl Penisprothesen-implantation |
| --- | --- | --- | --- |
| Chirurgische Klinik München-Bogenhausen | Urologie | München | 71 |
| Universitätsklinikum Freiburg | Klinik für Urologie | Freiburg | 32 |
| Agaplesion Markus Krankenhaus | Urologische Klinik | Frankfurt am Main | 30 |
| Ammerland-Klinik Westerstede | Klinik für Urologie und Kinderurologie | Westerstede | 29 |
| Universitätsklinikum Schleswig-Holstein, Campus Kiel | Klinik für Urologie und Kinderurologie | Kiel | 27 |
| Paracelsus Klinik Düsseldorf Golzheim | Urologie | Düsseldorf | 22 |
| Krankenhaus St. Franziskus | Klinik für Urologie | Mönchengladbach | 21 |
| HELIOS Klinikum Schwelm | Klinik für Urologie und Kinderurologie | Schwelm | 14 |
| Universitätsklinikum Köln | Klinik für Urologie, Uro-Onkologie, spezielle urologische und roboter-assistierte Chirurgie | Köln | 13 |
| Asklepios Westklinikum Hamburg GmbH | Abteilung für Urologie | Hamburg | 11 |
| Universitätsklinikum Hamburg-Eppendorf | Klinik und Poliklinik für Urologie | Hamburg | 10 |
| Klinikum rechts der Isar der Technischen Universität München | Klinik und Poliklinik für Urologie | München | 9 |
| Charité - Universitätsmedizin Berlin | Klinik für Urologie | Berlin | 8 |
| Universitätsklinikum Essen | Klinik für Urologie | Essen | 7 |
| Klinikum Leverkusen gGmbH | Klinik für Urologie | Leverkusen | 6 |
| Universitätsklinikum Münster | Centrum für Reproduktionsmedizin und Andrologie | Münster | 6 |
| Universitätsklinikum Tübingen | Universitätsklinik für Urologie | Tübingen | 6 |
| Asklepios Klinik Pasewalk | Klinik für Urologie | Pasewalk | 6 |
| Medizinische Hochschule Hannover | Klinik für Urologie und Urologische Onkologie | Hannover | 5 |
| Städtisches Klinikum Lüneburg gemeinnützige GmbH | Klinik für Urologie | Lüneburg | 5 |
| St. Antonius-Hospital Gronau | Klinik für Urologie, Kinderurologie und Urologische Onkologie | Gronau | 5 |
| Vivantes Humboldt-Klinikum | Klinik für Urologie | Berlin | 5 |
| Universitätsklinikum Leipzig AöR | Klinik und Poliklinik für Urologie | Leipzig | 5 |
| Universitätsmedizin Göttingen | Klinik für Urologie | Göttingen | 4 |
| KRH Klinikum Robert Koch Gehrden | Klinik für Urologie | Gehrden | 4 |
| Universitätsklinikum Bonn | Klinik und Poliklinik für Urologie und Kinderurologie | Bonn | 4 |
| Caritas-Krankenhaus St. Josef | Klinik für Urologie | Regensburg | 4 |
| Universitätsklinikum des Saarlandes | Klinik für Urologie und Kinderurologie | Homburg | 4 |
| Klinikum Ernst von Bergmann gemeinnützige GmbH | Klinik für Urologie | Potsdam | 4 |
| Klinikum Osnabrück GmbH | Klinik für Urologie und Kinderurologie | Osnabrück | 2 |
| Rottal-Inn Kliniken Kommunalunternehmen | Urologie | Eggenfelden | 2 |
| St. Hedwig-Krankenhaus Berlin | Klinik für Urologie | Berlin | 2 |
| Uinversitätsklinikum Schleswig-Holstein, Campus Lübeck | Klinik für Urologie | Lübeck | 1 |
| Asklepios Klinik Altona | Urologie | Hamburg | 1 |
| Asklepios Klinikum Harburg | Urologie | Hamburg | 1 |
| St. Bernward Krankenhaus | Urologische Klinik | Hildesheim | 1 |
| Helios Kliniken Mittelweser | Urologie | Nienburg | 1 |
| Helios Klinik Cuxhaven | Klinik für Urologie und Kinderurologie | Cuxhaven | 1 |
| Universitätsklinikum Düsseldorf | Klinik für Urologie | Düsseldorf | 1 |
| Helios St. Marien Klinik | Klinik für Urologie, Kinderurologie und urologische Onkologie | Duisburg | 1 |
| Evangelisches Klinikum Niederrhein | Klinik für Urologie | Oberhausen | 1 |
| HELIOS Klinikum Krefeld | Klinik für Urologie und Kinderurologie | Krefeld | 1 |
| Malteser Krankenhaus St. Josefshospital Uerdingen | Urologie und Kinderurologie | Krefeld | 1 |
| Städtische Kliniken Mönchengladbach GmbH | Klinik für Urologie und Kinderurologie | Mönchengladbach | 1 |
| Städtisches Klinikum Solingen | Klinik für Urologie und Kinderurologie | Solingen | 1 |
| HELIOS Klinikum Niederberg | Klinik für Urologie und Nephrologie | Velbert | 1 |
| GFO Kliniken Troisdorf, Betriebsstätte St. Josef Troisdorf | Urologie, St. Josef | Troisdorf | 1 |
| Rhein-Maas-Klinikum, Betriebsteil Bardenberg | Urologie und Kinderurologie | Würselen-Bardenberg | 1 |
| Klinikum Westmünsterland St. Marien- Krankenhaus Ahaus | Urologie und Kinderurologie | Ahaus | 1 |
| Prosper-Hospital | Klinik für Urologie & Zentrum für Minimalinvasive und Robotische Chirurgie in der Urologie | Recklinghausen | 1 |
| St. Elisabeth-Krankenhaus | Urologie | Ibbenbüren | 1 |
| Augusta-Kranken-Anstalt | Urologie | Bochum | 1 |
| Klinikum Dortmund Nord | Urologische Klinik | Dortmund | 1 |
| Marien Hospital Herne, Klinikum der Ruhr-Universität Bochum | Urologische Klinik | Herne | 1 |
| St. Josef-Krankenhaus, Hamm Bockum-Hövel | Klinik für Urologie, Kinderurologie und Uro-Gynäkologie | Hamm | 1 |
| Klinikum Darmstadt | Urologische Klinik | Darmstadt | 1 |
| Universitätsklinikum Gießen und Marburg, Standort Gießen | Klinik und Poliklinik für Urologie, Kinderurologie und Andrologie | Gießen | 1 |
| Kliniken des Main-Taunus-Kreises | Urologie | Bad Soden | 1 |
| Werner Wicker Klinik | Abteilung für Neuro-Urologie | Bad Wildungen | 1 |
| GPR Klinikum | Klinik für Urologie, Kinderurologie und onkologische Urologie | Rüsselsheim | 1 |
| Klinikum der Stadt Ludwigshafen am Rhein | Urologische Klinik | Ludwigshafen | 1 |
| Universitätsmedizin der Johannes Gutenberg-Universität Mainz | Klinik und Poliklinik für Urologie und Kinderurologie | Mainz | 1 |
| Diakonie-Klinikum Stuttgart | Urologische Klinik | Stuttgart | 1 |
| Caritas-Krankenhaus Bad Mergentheim | Urologie | Bad Mergentheim | 1 |
| Klinikum Mittelbaden Baden-Baden Balg | Klinik für Urologie | Baden-Baden | 1 |
| Universitätsklinikum Heidelberg | Urologische Klinik | Heidelberg | 1 |
| GRN Gesundheitszentren Rhein-Neckar, Kreiskrankenhaus Eberbach | Urologie | Eberbach | 1 |
| Siloah St. Trudpert Klinikum | Klinik für Urologie | Pforzheim | 1 |
| Ortenau Klinikum Offenburg-Gengenbach Standort Ebertplatz | Urologie und Kinderurologie | Offenburg | 1 |
| Klinikum am Steinenberg | Urologische Klinik | Reutlingen | 1 |
| Universitätsklinikum Ulm | Klinik für Urologie und Kinderurologie | Ulm | 1 |
| SRH Krankenhaus Sigmaringen | Urologie | Sigmaringen | 1 |
| Urologische Klinik München - Planegg | Urologie | Planegg | 1 |
| Klinikum Ingolstadt | Urologie | Ingolstadt | 1 |
| Klinikum der Universität München | Urologische Klinik und Poliklinik | München | 1 |
| Klinikum Landshut | Klinik für Urologie | Landshut | 1 |
| Donau Isar Klinikum Deggendorf | Klinik für Urologie und Kinderurologie | Deggendorf | 1 |
| Kliniken Nordoberpfalz, Klinikum Weiden | Klinik für Urologie | Weiden i. d. OPf. | 1 |
| Klinikum Bamberg - Betriebsstätte am Bruderwald | Klinik für Urologie und Kinderurologie | Bamberg | 1 |
| Klinikum Kulmbach | Urologie | Kulmbach | 1 |
| Klinikum Fürth | Klinik für Urologie und Kinderurologie | Fürth | 1 |
| Klinikum Nürnberg Nord | Klinik für Urologie | Nürnberg | 1 |
| Krankenhaus Martha-Maria Nürnberg | Urologisches Zentrum | Nürnberg | 1 |
| Missionsärztliche Klinik | Urologie | Würzburg | 1 |
| Helios Klinikum Berlin-Buch | Urologie | Berlin | 1 |
| Evangelisches Krankenhaus Königin Elisabeth Herzberge | Urologie | Berlin | 1 |
| Vivantes Klinikum Am Urban | Klinik für Urologie | Berlin | 1 |
| Universitätsmedizin Greifswald | Klinik und Poliklinik für Urologie | Greifswald | 1 |
| Universitätsmedizin Rostock | Urologische Klinik und Poliklinik | Rostock | 1 |
| Klinikum St. Georg | Klinik für Urologie und Andrologie | Leipzig | 1 |
| Lausitzer Seenland Klinikum | Klinik für Urologie, Kinderurologie und onkologische Urologie | Hoyerswerda | 1 |
| Sana Kliniken Leipziger Land, Klinikum Borna | Klinik für Urologie | Borna | 1 |
| HELIOS Klinikum Aue | Urologie und Kinderurologie | Aue | 1 |
| Klinikum Magdeburg | Klinik für Urologie und Kinderurologie | Magdeburg | 1 |
| Krankenhaus Martha-Maria Halle-Dölau | Klinik für Urologie, Kinderurologie und urologische Onkologie | Halle (Saale) | 1 |
| Südharz Klinikum Nordhausen | Klinik für Urologie | Nordhausen | 1 |
| HELIOS Klinikum Erfurt | Urologie | Erfurt | 1 |

**Ergänzende Tabelle 2**: Fallzahl der Penisprothesenimplantationen 2016 in urologischen Kliniken in Deutschland.
